# Ischemic Necrosis of Bilateral Second Toes in the Absence of Macrovascular Disease or Primary Vasculitis: A Unique Case of Systemic Sclerosis Sine Scleroderma (ssSSc)

**DOI:** 10.7759/cureus.94836

**Published:** 2025-10-18

**Authors:** Moamen Elhaddad, Maged Elhaddad, Valeria Corral, Tayler F Gant, Behnam David Massaband

**Affiliations:** 1 Orthopaedics, Cedars-Sinai Medical Center, Los Angeles, USA; 2 Internal Medicine, MedStar Health, Baltimore, USA; 3 Podiatry, Cedars-Sinai Medical Center, Los Angeles, USA; 4 Pathology and Laboratory Medicine, Cedars-Sinai Medical Center, Los Angeles, USA; 5 Podiatric Foot and Ankle Surgery, Cedars-Sinai Medical Center, Los Angeles, USA

**Keywords:** anticentromere antibodies, digital ischemia, systemic sclerosis sine scleroderma (ssssc), thrombotic vasculopathy, toe necrosis

## Abstract

Systemic sclerosis sine scleroderma (ssSSc) is an uncommon subset of systemic sclerosis, characterized by internal organ involvement and autoimmune serologies in the absence of skin thickening. Diagnosis is frequently delayed due to the lack of cutaneous findings, particularly when vascular manifestations precede classical rheumatologic features.

We report a compelling case of a 51-year-old woman who developed bilateral plantar second-toe ischemic necrosis despite having no diabetes, macrovascular disease, or known vasculitis. Extensive vascular imaging, including arterial duplex, ankle-brachial indices (ABIs), and toe photoplethysmography (PPG), was unremarkable. Serologic testing revealed a high-titer, centromere-pattern antinuclear antibody (ANA), and histopathology demonstrated thrombotic vasculopathy without features diagnostic of immune complex vasculitis. Nailfold capillaroscopy showed dilated capillary loops, and rheumatologic evaluation confirmed the diagnosis of ssSSc. The patient was treated conservatively with aspirin, nifedipine, and local wound care, resulting in complete epithelialization within eight weeks. This case highlights the importance of considering ssSSc in the differential diagnosis of unexplained digital ischemia - particularly in the lower extremities - and underscores the value of multidisciplinary collaboration in identifying rare autoimmune vascular syndromes presenting with limb-threatening ischemia.

## Introduction

Systemic sclerosis (SSc) is a rare, complex autoimmune connective tissue disease characterized by widespread microvascular dysfunction, immune dysregulation, and progressive fibrosis affecting the skin and internal organs [1]. Its estimated prevalence ranges from 7 to 33 cases per 100,000 individuals in Europe and North America [2]. The disease disproportionately affects women, with a female-to-male ratio of approximately 6:1 [2]. Traditionally, SSc has been classified into diffuse and limited cutaneous subsets, based on the extent of skin involvement [3]. However, a lesser-known variant - systemic sclerosis sine scleroderma (ssSSc) - accounts for approximately 10% of cases and is characterized by the absence of skin thickening despite internal organ involvement and hallmark autoantibodies [4,5]. Due to the lack of cutaneous clues, diagnosis of ssSSc is often delayed, and its manifestations may mimic other systemic or vascular disorders, particularly when initial symptoms are nonspecific.

Peripheral digital ischemia is one of the earliest and most disabling vascular manifestations of SSc, affecting approximately 30%-50% of patients during the course of the disease [6]. In rare cases, digital ischemia or gangrene may be the initial and sole manifestation, even preceding Raynaud’s phenomenon or overt signs of connective tissue disease [7,8]. This poses a diagnostic challenge, especially in the setting of normal peripheral vascular studies and the absence of classical vasculitis. In ssSSc, digital ulcers and ischemic changes are typically less severe than in other forms of SSc, but when present, they carry significant risk for tissue loss and functional disability [6].

We present a unique case of a 51-year-old woman who developed bilateral second-toe ischemic necrosis as the first sign of disease. This case is distinguished by the absence of systemic vasculitis, occlusive large-vessel disease, or skin sclerosis, yet fulfilled evolving criteria for ssSSc. The report highlights the value of histopathology, serologic markers, and careful rheumatologic assessment in recognizing this elusive presentation.

## Case presentation

A 51-year-old woman with a past medical history notable for Hashimoto's thyroiditis, fibromyalgia, myofascial pain syndrome, and joint hypermobility presented to the Emergency Department with sudden-onset discoloration and pain involving the plantar surfaces of both second toes. The symptoms developed acutely two months prior, following a seven-mile walk in Birkenstock sandals, initially presenting as a burning sensation and purplish discoloration that progressively darkened over several weeks into painful, sharply demarcated necrotic changes involving the distal pulp of the bilateral second toes. She had no prior history of diabetes, peripheral vascular disease, smoking, or known autoimmune vasculitis.

**Figure 1:**
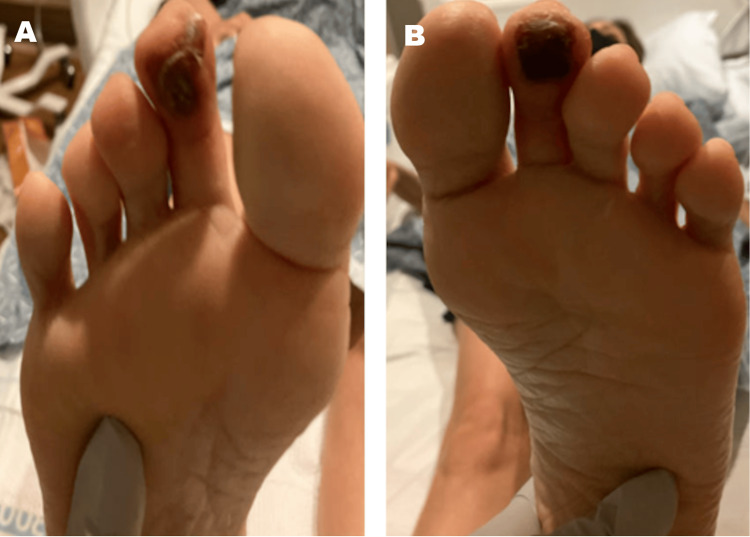
Bilateral Plantar Second Toe Necrosis at Initial Presentation Clinical photographs of the right (A) and left (B) feet showing symmetric, well-demarcated ischemic necrosis involving the distal pulp of the second toes. Both lesions are dry, non-ulcerated, and localized to the plantar surface, with preservation of surrounding soft tissue and no signs of infection or edema. This symmetric presentation, in the absence of trauma, infection, or large-vessel disease, was the key clinical finding that prompted an investigation for an underlying microvascular disorder.

On admission, physical examination revealed dry, darkened lesions localized to the plantar aspect of both second toes, without overlying cellulitis, edema, or signs of systemic infection (Figure 1). Pedal pulses were palpable, capillary refill time was brisk, and no skin thickening, sclerodactyly, or other cutaneous signs of SSc were initially observed. The patient was afebrile with stable vital signs throughout her hospitalization. Initial laboratory evaluation - including inflammatory markers, autoimmune serologies, complement levels, and vasculitis screening - is summarized in Table 1.

**Table 1:** Laboratory Results at Hospital Admission ANA, antinuclear antibody; ANCA, anti-neutrophil cytoplasmic antibody; CBC, complete blood count; CMP, comprehensive metabolic panel; CRP, C-reactive protein; dsDNA, double-stranded DNA; EIA, enzyme immunoassay; ENA, extractable nuclear antigen; ESR, erythrocyte sedimentation rate; IFA, indirect immunofluorescence assay; RF, rheumatoid factor; RNP, ribonucleoprotein; SSA/SSB, Sjögren’s-syndrome-related antigen A/B

Test	Result	Reference Range
ESR	11 mm/hr	< 20 mm/hr
CRP	< 0.5 mg/dL	< 1.0 mg/dL
CBC	Within normal limits	-
CMP	Within normal limits	-
RF	31 IU/mL	< 30 IU/mL
ANA (IFA)	1:640	< 1:40
Anti-dsDNA Antibody	<10 IU/mL	< 10 IU/mL
ENA RNP Antibody	2 U	< 20 U
ENA Smith (Sm) Antibody (EIA)	1 U	< 20 U
ENA SSA (Ro) Antibody (EIA)	3 U	< 20 U
ENA SSB (La) Antibody (EIA)	1 U	< 20 U
Scleroderma (Scl-70) Antibody	<1 U	< 7 U
Complement C4 (C4C)	30 mg/dL	15-57 mg/dL
Complement C3 (C3C)	115 mg/dL	83-193 mg/dL
ANCA	<10 titer	< 10

**Figure 2:**
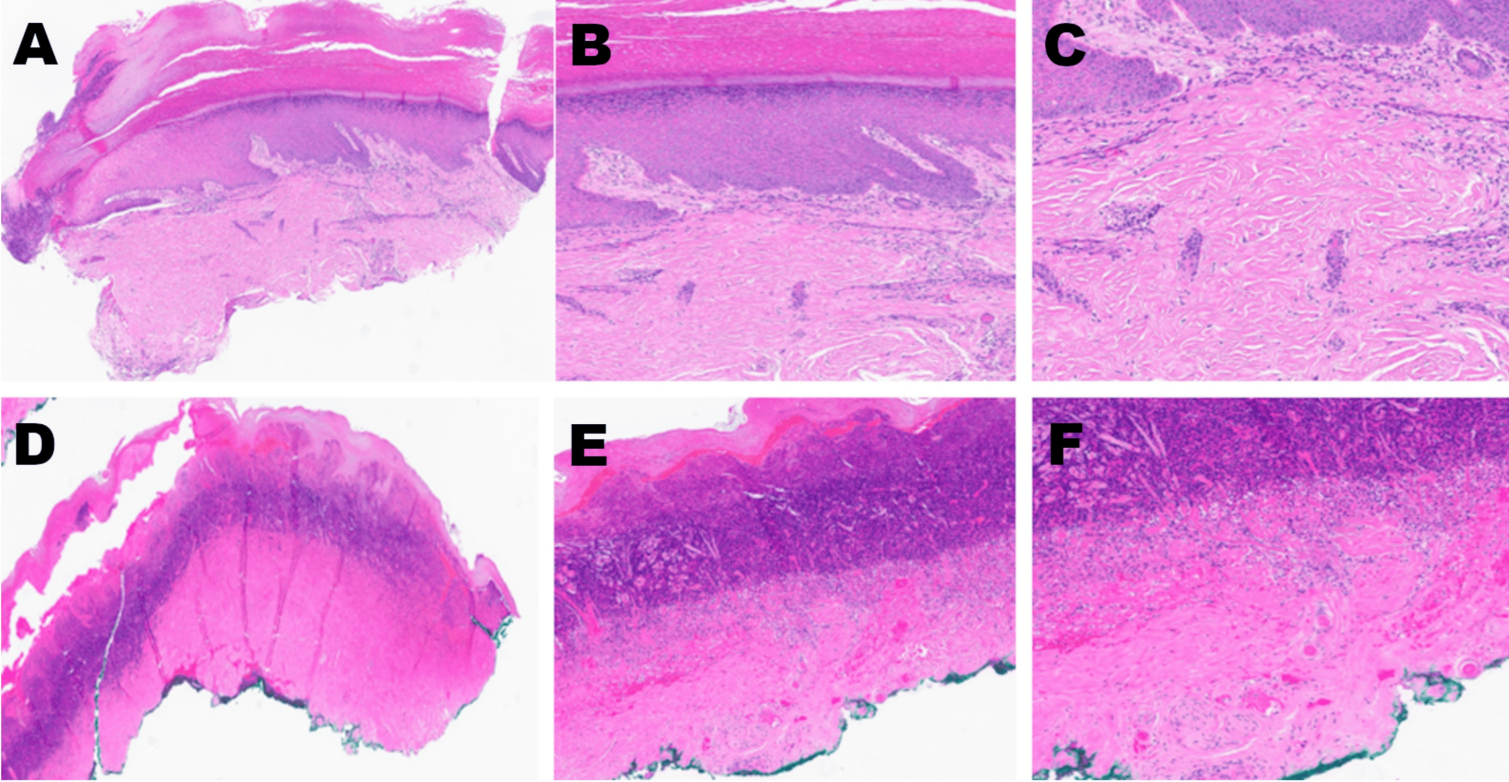
Histopathologic Features of the Second Toe Lesions (H&E Staining) Panels A-C (left toe): Low- to medium-power views (4×, 8×, 10×) showing mild superficial perivascular mixed inflammatory infiltrate with extravasated erythrocytes and scattered nuclear debris, consistent with early microvascular injury. Panels D-F (right toe): Serial magnifications (4×, 8×, 10×) revealing transepidermal necrosis, pandermal thrombotic vasculopathy with vascular occlusion, and prominent leukocytoclasis, without overt evidence of active vasculitis. These histopathologic findings are consistent with a thrombotic microangiopathy and were pivotal in ruling out primary vasculitis, thereby supporting the diagnosis of a systemic sclerosis-related vasculopathy.

A comprehensive vascular workup, including bilateral lower extremity arterial duplex ultrasonography, ankle-brachial indices (ABIs), toe photoplethysmography (PPG), and aortoiliac duplex studies, was entirely normal. No evidence of peripheral arterial disease (PAD), venous thrombosis, or embolic source was identified. Bilateral toe radiographs were unremarkable. Given the isolated and unexplained bilateral digital ischemia, dermatopathology consultation was pursued. Excisional biopsies from both second toes revealed transepidermal necrosis with pandermal thrombotic vasculopathy and leukocytoclasis (Figure 2). Direct immunofluorescence (DIF) of perilesional tissue was performed but did not reveal immune complex deposition, thereby ruling out classic small vessel vasculitis. Rheumatology assessed the patient and, in light of her high-titer antinuclear antibody (ANA) with centromere pattern, history of Raynaud’s phenomenon, nailfold capillary dilatation, solitary telangiectasia, and absence of sclerodermatous skin changes, diagnosed ssSSc. A summary of the key diagnostic findings is provided in Table 2.

**Table 2:** Summary of Key Diagnostic Findings Leading to the Diagnosis of Systemic Sclerosis Sine Scleroderma ANA, antinuclear antibody; ABI, ankle-brachial index

Domain	Key Finding
Clinical	Bilateral second-toe necrosis; History of Raynaud's phenomenon; Solitary telangiectasia; Absence of skin thickening.
Serologic	High-titer ANA with centromere pattern.
Imaging & Vascular Studies	Normal arterial duplex, ABI, and photoplethysmography; Nailfold capillaroscopy showing dilated capillary loops.
Histopathologic	Transepidermal necrosis with pandermal thrombotic vasculopathy; Negative direct immunofluorescence for immune complex deposition.

**Figure 3:**
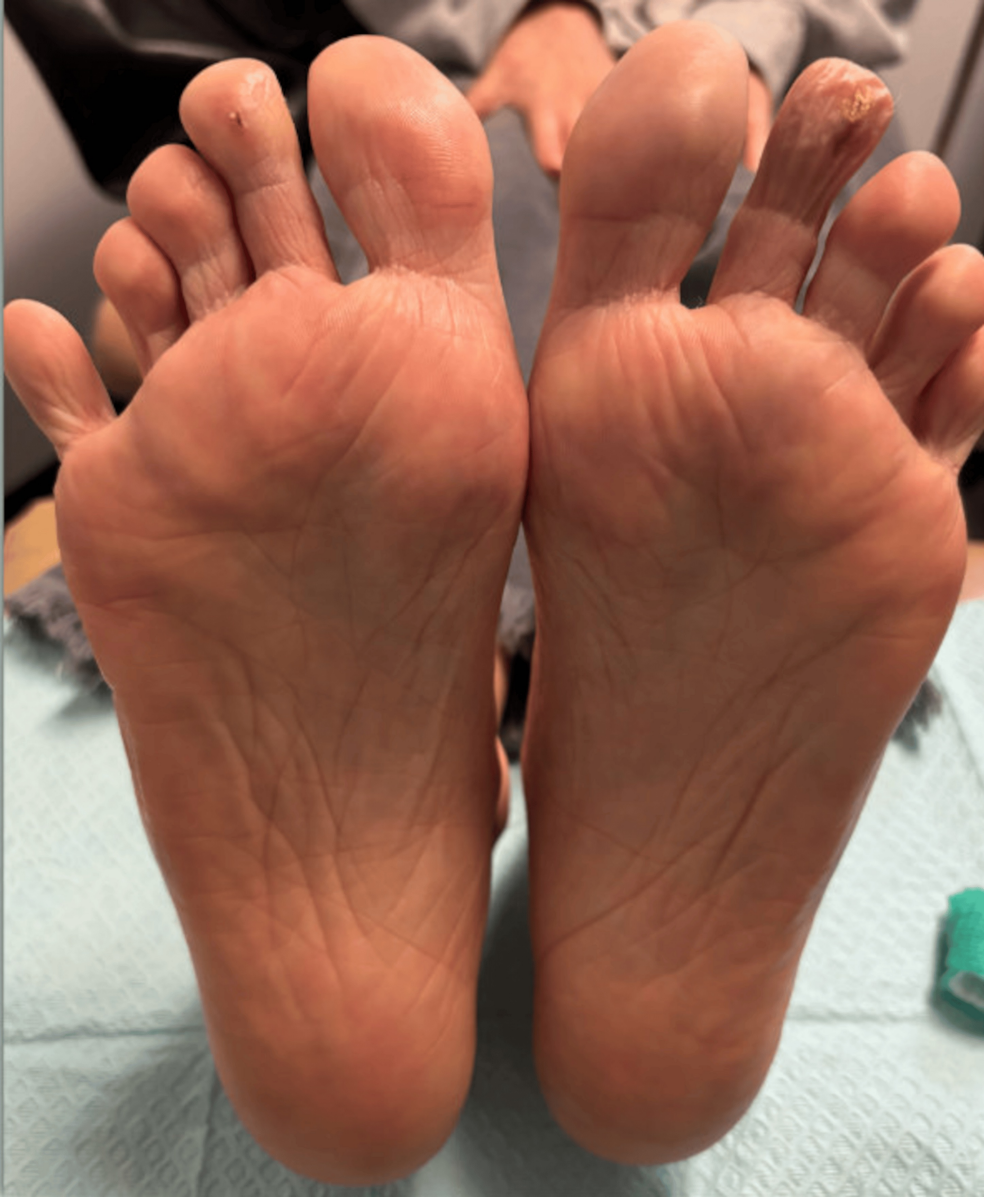
Healing Outcome of Bilateral Second-Toe Ischemia Near-complete epithelialization was observed at the eight-week follow-up. This successful healing, with conservative management, supports the diagnosis of a non-inflammatory vasculopathy and demonstrates a positive clinical outcome.

The patient was treated conservatively with daily aspirin (81 mg) and extended-release nifedipine, initially 30 mg and later titrated to 60 mg to enhance digital perfusion. Gabapentin was prescribed to alleviate neuropathic discomfort. Surgical intervention, including debridement or amputation, was deemed unnecessary. She was discharged with a home health nursing plan for wound care and scheduled outpatient follow-up with rheumatology and podiatry. Serial examinations demonstrated progressive healing of the biopsy sites without signs of superinfection or tissue deterioration. No evidence of systemic organ involvement emerged during the post-hospital course. At her eight-week follow-up, the patient reported complete epithelialization of the digital lesions and resolution of pain (Figure 3).

## Discussion

ssSSc is an uncommon subset of scleroderma that accounts for roughly 10% of SSc cases [4]. By definition, ssSSc patients lack the characteristic skin thickening of scleroderma, making diagnosis challenging in the absence of cutaneous clues [4]. Nevertheless, virtually all ssSSc patients develop Raynaud’s phenomenon - approximately 97% in one large cohort - and a significant minority experience digital ischemic lesions, such as ulcers or pitting scars, over time [4]. In our patient, the presentation of bilateral second-toe necrosis with no skin sclerosis or macrovascular disease highlights this diagnostic difficulty. It is exceedingly rare for SSc to manifest initially as isolated digital gangrene without other overt clinical features [7,8]. Indeed, previously reported cases emphasize the rarity of such an occurrence and underscore the need for a high index of suspicion when confronting digital ischemia of unclear origin.

Our case can be compared with a handful of similar reports in the literature. Sachsenberg-Studer et al. described four women who presented with severe digital necrosis, Raynaud’s phenomenon, and high-titer anticentromere antibodies in the absence of sclerodactyly or internal organ fibrosis [7]. They argued that this triad should be considered an atypical variant of SSc, distinct from classic scleroderma. Similarly, Bolster et al. reported a 53-year-old woman who developed spontaneous gangrene of a fingertip with no prior scleroderma features aside from a strongly positive anticentromere ANA [8]. In that case, as in ours, no diabetes, arteriosclerosis, or vasculitis was found, illustrating that an autoimmune vasculopathy can underlie digital ischemia even when connective tissue disease signs are subtle or absent [8]. From a rheumatologic perspective, larger studies also acknowledge such presentations. For example, one multicenter cohort noted that three of four patients identified with ssSSc had episodes of digital necrosis [9]. Likewise, patients with limited cutaneous SSc who carry anticentromere antibodies are known to be at heightened risk for critical digital ischemia [8]. On the whole, however, ssSSc patients tend to have fewer and less severe digital ulcers than those with cutaneous scleroderma [4], which underlines the unusual severity of our patient’s condition. Our patient's ANA titer of 1:640 is a high positive, significantly above the normal cutoff (<1:40), which strongly reinforces the autoimmune etiology and is consistent with the serological profile of ssSSc.

Histopathology and DIF played a key role in our patient’s diagnostic evaluation, helping to distinguish between an SSc-related vasculopathy and other etiologies. The biopsy of the necrotic toe demonstrated thrombotic vasculopathy with fibrinoid necrosis and leukocytoclastic debris, changes that raised concern for small-vessel vasculitis. DIF further revealed deposition of immunoreactants in the vessel walls, a finding that typically suggests immune complex-mediated leukocytoclastic vasculitis. It is important to interpret these findings in context. Biopsies taken from ulcerated or ischemic tissue often show “incidental” vasculitic changes that do not necessarily indicate a primary vasculitis [10]. The presence of thrombotic vasculopathy, without definitive immune complex deposition on DIF, is a key distinguishing feature from primary immune-complex mediated vasculitides (e.g., lupus or cryoglobulinemic vasculitis). Furthermore, the absence of an embolic source on vascular imaging and the lack of inflammatory markers help differentiate this from thromboembolic disease or a primary systemic vasculitis (e.g., anti-neutrophil cytoplasmic antibody (ANCA)-associated vasculitis). In SSc, the fundamental vascular pathology is a non-inflammatory obliterative microangiopathy, characterized by intimal proliferation and narrowing of arterioles (endarteritis obliterans), rather than an immune-complex vasculitis [11]. This SSc-associated vasculopathy is often termed “pauci-immune,” although, on occasion, a true vasculitis can overlap with SSc [12]. In our patient, the absence of ANCA, cryoglobulins, and antiphospholipid antibodies effectively ruled out primary small-vessel vasculitides. Combined with the presence of giant capillary loops on nailfold exam and a high-titer centromere-pattern ANA, the tissue findings were best interpreted as secondary to SSc microvascular injury (with possible minor immune complex trapping) rather than a separate systemic vasculitic disorder. The diagnosis of ssSSc was solidified by the explicit correlation of the clinical presentation (bilateral digital necrosis) with the serological hallmark (high-titer centromere ANA), the in-vivo evidence of microvascular disease (nailfold capillary dilatation), and the histopathologic correlate (thrombotic vasculopathy). Thus, the histology and DIF findings, while abnormal, served mainly to exclude other diagnoses and reinforce the conclusion of an SSc-related thrombotic vasculopathy.

A potential diagnostic limitation was the histopathologic overlap, as biopsies from ischemic tissue can show “incidental” vasculitic-like changes, making it challenging to definitively rule out a concurrent, low-grade inflammatory process. However, the overall clinical context and the patient's excellent response to purely vasodilatory therapy make ssSSc the most probable diagnosis.

This case carries important educational and clinical lessons for podiatric and multidisciplinary care. Podiatric physicians and wound care specialists are often the first to evaluate digital ulcerations or gangrene, and it is crucial that they consider underlying autoimmune causes when common etiologies have been excluded. In a patient without diabetes, atherosclerotic risk factors, or evidence of emboli, unexplained toe necrosis should prompt an evaluation for phenomena like Raynaud’s and for serological markers of connective tissue disease. Our patient’s work-up exemplifies this approach: subtle clues, such as a history of Raynaud’s, nailfold capillary dilation, and a centromere-pattern ANA, were pivotal in arriving at the correct diagnosis. Early collaboration with dermatology and rheumatology allowed these findings to be recognized, leading to appropriate management. Notably, the patient’s toes healed over about eight weeks with conservative wound care, low-dose aspirin, and the calcium-channel blocker nifedipine. This favorable outcome aligns with the recommended management of SSc-related digital ischemia, which centers on vascular support rather than immunosuppression. Vasodilators (e.g., dihydropyridine calcium blockers and prostacyclin analogs) and antiplatelet or anticoagulant therapy are mainstays for scleroderma digital ulcers [11], alongside meticulous wound care and risk factor modification, such as protecting from cold exposure and smoking cessation [11]. In contrast, aggressive immunosuppressive therapy is reserved for true inflammatory vasculitis or major organ involvement in SSc, which were not present in this case.

Finally, our case highlights that SSc, even without skin tightening, can significantly involve the lower extremities. Foot ulcers and pedal digital ischemia are an under-recognized problem in scleroderma patients. Studies have shown that about a quarter of SSc patients experience foot ulceration during their illness, and podiatric examinations reveal high frequencies of pedal lesions and deformities [13]. Such foot problems contribute substantially to patient morbidity and disability [13]. This highlights the need for multidisciplinary management: regular foot assessments, patient education on foot care, and prompt treatment of even minor digital lesions are important components of comprehensive SSc care. Our patient’s outcome illustrates that, with coordinated care - involving podiatry for wound management, rheumatology for systemic therapy, and dermatology for diagnostic skin examinations - even an unusual presentation of ssSSc can be correctly diagnosed and successfully managed. This case reinforces the importance of recognizing atypical peripheral ischemia as a potential early indicator of underlying systemic rheumatic disease. Maintaining a high index of suspicion for features of ssSSc in such presentations may be critical to preserving limb function and, in some instances, saving lives.

## Conclusions

This case illustrates a rare initial presentation in which ssSSc may present as symmetric digital toe necrosis, in the absence of cutaneous findings, vasculitis, or macrovascular disease. It emphasizes the diagnostic value of integrating nailfold capillaroscopy, high-titer centromere-pattern ANA, and histopathologic evaluation in cases of unexplained digital ischemia. Podiatric and multidisciplinary providers should maintain a high index of suspicion for occult autoimmune disease when common causes are excluded. Prompt recognition and conservative management with vasodilators, antiplatelet therapy, and local wound care led to complete healing. While this single case report demonstrates a successful outcome, it highlights the need for awareness of ssSSc in atypical digital ischemia to prevent delays in diagnosis and optimize outcomes.
